# Visual Attention, Behavioral Intention, and Choice Behavior Among Older Consumers Toward Sports Marketing Images: An Eye-Tracking Study

**DOI:** 10.3389/fpsyg.2022.855089

**Published:** 2022-05-19

**Authors:** Tsai-Chiao Wang, Ta-Wei Tang, Chia-Liang Tsai

**Affiliations:** ^1^Institute of Physical Education, Health and Leisure Studies, College of Management, National Cheng Kung University, Tainan City, Taiwan; ^2^Department of Leisure and Recreation Management, College of Management, Asia University, Taichung City, Taiwan

**Keywords:** behavioral intention, natural sportscape, urban sportscape, visual attention, eye movement

## Abstract

Mental health benefits (i.e., relaxing and relieving pressure) can influence consumers’ consumption decisions. However, there is still no clear understanding of the impact of mental health benefits on visual attention, behavioral intention, and choice behavior. Study 1 was thus aimed at exploring the visual attention and behavioral intention of older consumers with respect to exercise consumption. A sample of 186 older consumers was investigated. An eye-tracking analysis was used to evaluate the visual attention of participants observing health promotion messages, and questionnaires were used to assess the behavioral intention of the older consumers under consideration in this work. The findings confirmed that marketing pictures combining natural sportscapes with prevention focus messages (i.e., conveying information to consumers that it is safe and not easy to be injured when engaging in yoga activities in natural settings) can best capture older consumers’ visual attention (e.g., fixation numbers and fixation times) and behavioral intentions. In Study 2, 75 participants were recruited. It was found that marketing pictures combining natural sportscapes with prevention focus messages were selected more by the participants, with health communication images successfully attracting them to choose the sports program products being presented. The findings of the two studies suggested that marketing pictures can effectively stimulate consumers’ visual attention and has effects on their behavioral intention and choices toward exercising in a safe, natural environment.

## Introduction

As people increasingly become more responsible for their own health, designing effective online health messages is becoming more important than in the past (Yoshida, 2017). Although older adults are undergoing an aging process, they may need or have the motivation to improve their wellbeing through exercise (Penedo and Dahn, 2005; Thøgersen-Ntoumani et al., 2012). However, previous sports marketing research was focused mainly on young adults, rather than on older people. Thus, researchers still do not know enough about the development of online sports marketing advertising that can attract older adults to engage in exercise (Thøgersen-Ntoumani et al., 2012). Therefore, the development of online sports marketing images that can capture the visual attention of older adults and influence their subsequent behavioral intentions and choices is a key issue.

The features of a sportscape (illustration) (Calogiuri and Elliott, 2017) and a message (text) (Pfeffer, 2013) are two crucial variables influencing older consumers’ engagement in exercise. First, a sportscape is defined as a landscape in which people engage in exercise (Bitner, 1992). Sportscapes can be separated into natural and urban types. A natural sportscape refers to a place in nature where it is suitable for older consumers to engage in exercise. An urban sportscape refers to an indoor space that can be used for exercise, including sports centers or gyms (Calogiuri and Elliott, 2017). Natural sportscapes and urban sportscapes are both useful environments for promoting physical activity (Calogiuri and Elliott, 2017). Second, through a sports marketing message, sports firms can convey the goal of healthy living to older consumers. Regulatory focus theory is typically used for consumer research because it can explain various consumer decision-making behaviors (Aaker and Lee, 2001). When pursuing a goal, the decisions made by an individual will be affected by the type of messages they receive and the focus of those messages (Higgins, 1997). The regulatory focus theory argues that consumers differ in the way they look at their goals and thus adopt different approaches to achieving them (Higgins, 1997; Avnet and Higgins, 2006). Specifically, the regulatory focus theory suggests two independent regulatory focus messages: promotion- and prevention-focused messages, which provide effective persuasion strategies by which customers can pursue their goals (Lee and Aaker, 2004). Promotion-focused messages focus on hope, achievement, and progressive demand. In contrast, prevention focus messages focus on safety, responsibility, and safety needs (Aaker and Lee, 2001; Lee and Aaker, 2004).

To ensure that messages are effectively accepted, one must first consider the characteristics of the information recipients and use that data to develop information that is persuasive to them. Therefore, marketing advertisements must first attract the visual attention of older adults before they have the opportunity to stimulate subsequent behavioral intentions and in turn influence their choices (Kahn, 2017). Attention is a selective mechanism for determining the extent to which a customer is concerned with a specific stimulus (Solomon et al., 2013). Customers do not treat all marketing images the same way (Engelke and Le Callet, 2015), and they assign more visual attention to features they are interested in. Behavioral intention is defined as “the degree to which a person has formulated conscious plans to perform or not to perform some specified future behavior” (Westerbeek and Shilbury, 2003). Although behavioral intention does not always lead to real behavior, a stronger degree of intention leads to a greater possibility of engaging in a specific behavior (Ajzen, 1991), for example purchasing behavior (Ramsøy et al., 2019). Visual images can reduce cognitive effort and allow customers to process information faster (Kahn, 2017), thereby helping customers choose a specific exercise program more easily.

There are few studies in the sports marketing field that have used neurophysiological techniques to explore memories and purchase intentions after subjects watch sponsored advertisements (i.e., Dos Santos et al., 2019). In addition, to the best of our knowledge, limited studies have focused on consumer responses to online advertisements provided by sports firms. This implies that sports marketing researchers know little about the actual level of attention individuals pay toward such messages. In addition, previous researchers argue that sports firms still rely on experience or self-reporting when designing and evaluating health information (i.e., Yoshida, 2017). Since eye tracking goes beyond self-reported data, it provides a fairly objective measure and shows the process by which individuals deal with visual and textual information (Aaker and Lee, 2001). To promote the marketing effectiveness of online exercise courses, in this study, an eye tracking technique was used to analyze older consumers’ visual, psychological, and choice responses after they observe online exercise advertisements.

In summary, the purpose of this research is to evaluate customers’ visual attention, behavioral intention, and choice responses toward online sports marketing advertising. The combination of eye tracking technology and surveys on customer exercise choices provides industry practitioners with a unique strategy by which to observe the process and results of individual behavior that occurs at the time of purchase (Krucien et al., 2017). Accordingly, the researchers compare the effectiveness of various exercise marketing pictures in this study, adding insights that may contribute to the effectiveness of sports marketing designs.

## Literature Review and Hypotheses Development

### The Natural Environment and Stress Reduction Theory

The theory of stress reduction is a useful theory to explain why contact with nature can reduce stress (Bratman et al., 2019; Whitburn et al., 2019). Exposure to the natural environment initiates an innate, rapid, and emotionally driven process, thereby reducing physical and psychological stress (Ulrich et al., 1991; Bratman et al., 2019; Whitburn et al., 2019). Thompson Coon et al. (2011) compared the physical and mental health of people who participate in physical activities in a natural outdoor environment with those who participate in indoor physical activities. They found that compared with exercising indoors, exercising in a natural environment brings more positive psychological responses, including reduced stress and lower levels of frustration.

### Older Adults and Regulatory Focus Theory

Previous studies have confirmed that people’s preferences for advertising are actually a reflection of their goals (Clary et al., 1994; Han and Shavitt, 1994; Lavine and Snyder, 1996). In particular, older adults emphasize prevention goals because they feel that their time in the future is limited (Micu and Chowdhury, 2010). Older adults will expect to suffer more losses in the future than young adults (Heckhausen and Krueger, 1993), and they mention more goals related to avoiding losses, such as preventing physical injuries. Therefore, older adults may be driven mainly by goals related to prevention rather than goals related to promotion (Micu and Chowdhury, 2010).

### Older Consumers’ Motivations for Exercising and Preferences for Marketing Images Designs

In terms of sportscapes, Calogiuri and Elliott (2017) analyzed older consumers’ motivations for exercising in natural or urban sportscapes. The results of their research showed that older consumers are comparatively more motivated by convenience and natural experiences and less motivated by the body-oriented and social-related factors that typically motivate younger consumers (Calogiuri and Elliott, 2017). For older consumers, exercising in a comfortable, safe natural environment is more beneficial to their health (Mackay and Neill, 2010). Further, in terms of text, regulatory focus theory is a useful framework for developing sports marketing messages. The regulatory focus theory (including promotion-focused messages and prevention-focused messages) has been used to develop two different types of advertising messages intended to encourage active lifestyles. Promotion-focused messages highlight the meaning of pursuing health through exercise and emphasize the results of the pursuit of physical fitness (i.e., doing yoga helps enhance the memory of older consumers). In contrast, prevention-focused messages highlight the safety issues related to engaging in exercise (Aaker and Lee, 2001; Lee and Aaker, 2004) and emphasize information related to exercise safety (i.e., engaging in yoga is safe for older consumers).

### Neuromarketing and Eye Tracking

Neuromarketing is essentially the application of neuroscience methods to understand consumer psychology and predict decision-making behavior (Ramsøy et al., 2019). In the study of consumer behavior, these widely used neuroscience tools and methods are improving our understanding of preference formation and online choices (Bol et al., 2016; Ramsøy et al., 2019). While fMRI has been a dominant methodology in consumer neuroscience, one of the most useful tools for understanding visual attention is eye-tracking (Ramsøy et al., 2019). Eye tracking has been widely used in the field of industrial and academic marketing research to measure consumer attention to advertisements on web pages, product packaging labels, and point-of-purchase displays. Leading multinational companies and consumer goods manufacturers (e.g., Unilever, Procter and Gamble, and Pepsi) often use eye tracking technology to measure the effectiveness of a series of marketing stimuli and utilize the visual attention of consumers to predict their subsequent purchase decisions (Wedel and Pieters, 2012; Harris et al., 2018; Ramsøy et al., 2019).

In the field of marketing research, eye tracking technology that captures individual eye movement trails has been used to evaluate visual attention to advertising (Wedel and Pieters, 2012) and subsequent choice decisions, but few applications appear in the sports industry literature (Discombe and Cotterill, 2015). Since eye movement data can help researchers gain insights into how individuals deal with online health messages (e.g., text or visual messages, or both) (Bol et al., 2016), in this study, eye tracking is thus used to identify how older consumers process messages (i.e., text and/or illustrations) and to examine the effectiveness of these messages in terms of predicting future purchase choices.

### Yoga as a Suitable Exercise for Older Adults

Older consumers are often unable to sustain high-intensity exercise due to the deterioration of their physical functions. However, yoga is a low-intensity exercise that has effects including stabilizing the autonomic nervous system, reducing stress, and eliminating mental pressure so as to achieve spiritual and emotional stability (Black et al., 2013). Thus, when compared with many other forms of exercise, yoga is safe and suitable for older consumers and patients with chronic conditions (Desveaux et al., 2015). Many studies also confirm that yoga can bring physical and psychological benefits to older consumers. Yoga can effectively improve the mood of older consumers (reducing depression, stress, and dispiritedness, and boosting mood), as well as their cognitive functions (i.e., attention, memory, and resiliency) (Black et al., 2013; Desveaux et al., 2015).

### Relationships Between a Sportscape and Visual Attention, Behavioral Intention, and Choices

The physical environment plays a key role in the way that older consumers experience sports services. Funk (2017) suggested that when assessing the quality of an exercise field, the sports user perspective should be considered because a sportscape will not be able to operate in an effective manner until the needs of the target user are met. The motivations leading sports users to engage in exercise can reflect the sportscape they prefer. While exercising in a natural environment, older consumers can enjoy the scenery (e.g., sky, woods, and grass) (Calogiuri and Elliott, 2017). Engaging in exercise in a natural environment helps restore people’s health and focus (Hug et al., 2009). In contrast, urban sportscapes are designed in enclosed spaces. In these spaces, exercisers can observe their physical appearance and movements in mirrors or reference the images displayed on exercise machines while exercising (Calogiuri and Elliott, 2017). In fact, the motivation of older consumers to exercise may also be related to their sportscape preferences. Research on the stress reduction theory has shown that briefly exercising in a natural environment can relieve fatigue and stress (Ulrich et al., 1991). Exercise in a natural environment (e.g., walking in a forest) can easily reduce an individual’s stress and stimulate that person’s desire to be close to nature, providing the individual with an opportunity for mental and physical stress reduction (Ulrich et al., 1991). In contrast, indoor exercise environments do not provide older consumers with as much “walking-out” stimuli that create opportunities for recovering either physically or psychologically (Ulrich et al., 1991; Bratman et al., 2019; Whitburn et al., 2019).

In addition to making older consumers feel relaxed, natural sportscapes also make them feel safer than urban sportscapes. Older consumers feel more comfortable and secure in natural sportscapes where they can regulate their exercise intensity (Calogiuri and Elliott, 2017). Physical exercise in a natural sportscape has been found to be positively associated with a reduction in the degree of risk of physical weakness. Thus, older consumers will choose to be close to nature because it satisfies their need for both natural experiences as well as convenience. Therefore, individuals may prefer to observe sportscapes images featuring natural environments that can satisfy these needs. These natural sportscapes will further stimulate their behavioral intentions and choices to exercise in sportscapes. Thus, natural sportscapes images may be more attractive to the older consumers and thus gain higher visual attention, which may lead to behavioral intentions and more purchase choices than urban sportscapes images. The following hypotheses were formulated accordingly:

H1: Images of natural sportscapes attract the visual attention of older customers more than images of urban sportscapes.

H2: Images of natural sportscapes lead to more behavioral intention in older customers than images of urban sportscapes.

H3: Images of natural sportscapes lead to more purchase choices in older customers than images of urban sportscapes.

### Relationships Between Regulatory Focus Messages and Visual Attention and Behavioral Intention

Promotion-focused messages emphasize the health outcomes brought about by exercise, while prevention-focused messages highlight the safety of exercise and avoidance of injuries during exercise (Aaker and Lee, 2001; Lee and Aaker, 2004). Individuals will pay more visual attention to information that can meet their needs, so the induced behavioral intention will be higher. Older consumers are not concerned as much with their overall physical appearance as they are about engaging in a gentle form of exercise (Thøgersen-Ntoumani et al., 2012). They prefer to adjust the intensity of their exercise according to their own assessment of their physical condition (Samra et al., 2019). By exercising in a natural environment (such as on grass or wooden floors), older consumers can exercise freely without fear of injury (Mackay and Neill, 2010; Calogiuri and Elliott, 2017). Therefore, it is assumed here that prevention-focused messages will be more effective than promotion-focused messages in terms of attracting older consumers’ visual attention and promoting their behavioral intention toward online exercise consumption. The following hypotheses are thus proposed:

H4: Images of exercise in a natural sportscape with a prevention-focused message attract the visual attention of older customers more than images of exercise in a natural sportscape with a promotion-focused message.

H5: Images of exercise in a natural sportscape with a prevention-focused message enhance the behavioral intention of older customers more than images of exercise in a natural sportscape with a promotion-focused message.

### Relationships Between Regulatory-Focused Messages and Purchase Choices

As individuals age, the likelihood of loss becomes more salient. Older adults may prefer a prevention focus more than younger adults when making decisions (Micu and Chowdhury, 2010). Based on the theory of regulatory relevance, marketing information that responds to consumer concerns will induce more favorable attitudes (Avnet and Higgins, 2006). When people make decisions or choices based on their motivational goals, they will “feel right” about marketing information that meets their desired goals. Then, this “feel right” experience will be further transferred to follow-up choices (Avnet and Higgins, 2006).

Compared with young people, older adults tend to think that they will suffer from more losses in their future lives (Heckhausen and Krueger, 1993). Therefore, they tend to adopt more activities and choices that can help avoid losses in both their current and future life. The following hypotheses are thus proposed:

H6: Images of exercise in a natural sportscape with a prevention-focused message enhance the purchase choice of older customers more than images of exercise in a natural sportscape with a promotion-focused message.

**FIGURE 1:**
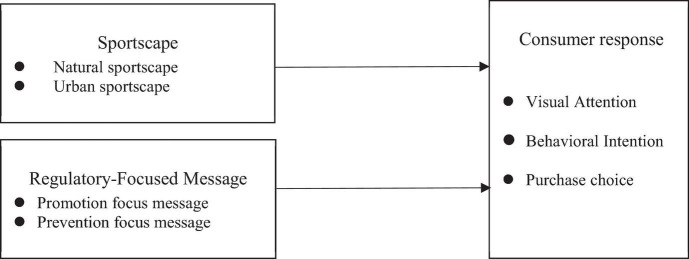
Research framework.

The research framework mentioned above is illustrated in Figure 1.

## Study 1

### Methods

#### Participants

The researchers contacted seniors (age > 50 years) in the Taichung Senior Citizens Association database in Taiwan through flyers or personal visits and invited them to participate in the experiment. In addition, the researchers also recruited older participants by snowballing. The criteria for selecting participants were as follows: (1) Individuals were required to be over the age of 50. (2) The participants had normal vision in both eyes (more than 0.8 after correction) and no major eye diseases (e.g., macular lesions). (3) When selecting the participants, the researchers excluded older consumers who could not physically move.

This study was approved by and carried out in accordance with the recommendations of National Cheng Kung University’s Ethics Committee, and written informed consent was obtained from all participants in accordance with the Declaration of Helsinki.

#### Research Design

There were four experimental conditions (see Table 1): two sportscapes (a natural sportscape and an urban sportscape) and two regulatory-focused messages (a promotion-focused message and a prevention-focused message). There were 16 images used in total, including four pictures for each variable. These images were obtained from Freepik.com (a website providing free resources for personal and commercial purpose with attribution) and were re-designed for this study. Each picture had a similar background. Previous studies have indicated that an eye tracker can be used to collect data related to people’s visual attention (Wang et al., 2018, 2019a). Human visual attention typically lasts for 200–500 ms (Wedel and Pieters, 2012), so values below 200 ms were excluded. The researchers used the following methods to analyze visual attention: A heat map showed the position and density of the visual focus (gaze point) when the participant looked at an image, where the red area in the heat map indicated longer or more frequent fixation, and the green area indicated a shorter or less frequent fixation. Fixation duration and counts were used to assess visual attention in this work (Wang and Sparks, 2016), where the fixation duration indicated the time the observer spent on one fixation, and the fixation count indicated the number of times the observer’s eye movement stayed on an area of interest in the picture (Engelke and Le Callet, 2015). The gaze plot showed the sequence of the participant’s fixations on the various AOIs when the participant looked at a given picture (Engelke and Le Callet, 2015). The observation sequence indicated the information that the participants were most interested in.

**TABLE 1:** Experimental conditions.

Factors		Regulatory focus messages	
		Promotion focus message	Prevention focus message
		**A condition:**	**B condition:**
Sportscapes	Natural sportscape	Natural sportscape and Promotion- focused message	Natural sportscape and Prevention-focused message
		**C condition:**	**D condition:**
	Urban sportscape	Urban sportscape and Promotion-focused message	Urban sportscape and Prevention-focused message

In addition to eye-tracking, questionnaires were also used to assess older consumers’ behavioral intention toward participating in yoga. The behavioral intention questionnaire had a total of five questions. It was a modified version of the questionnaire used by Zeithaml et al. (1996). The questions were measured on a 5-point Likert scale. The participants could respond on a scale ranging from 1 to 5, with 1 indicating strong disagreement and 5 indicating strong agreement. The items included: (1) I would like to know more about the health benefits of yoga. (2) This image helps promote yoga. (3) I want to see more yoga-related images. (4) This picture made me want to practice yoga, and (5) I will say a positive thing of yoga to others. Cronbach’s Alpha of the behavioral intention is 0.88. This study also verified the three indicators suggested by Hair et al. (2010), to confirm the reliability and validity of the scale of behavioral intention. First, the factor loadings of all measured variables were significant, greater than 0.7, and exceeded the acceptable level suggested by Hair et al. (2010). Second, the construct reliability of behavioral intention is 0.92, which exceeded the 0.7 level suggested by Hair et al. (2010). Third, the average variance extracted of behavioral intention is 0.68, which exceeded the 0.5 level suggested by Fornell and Larcker (1981), indicating that the variables had good convergent validity. Those results indicating that the variables had good reliability and validity.

In this study, a single yoga posture was used as the controllable factor in the experimental pictures. In addition, different sports scenes (natural sportscape vs. urban sportscape) were used in the backgrounds of the images, and regulatory-focused messages (promotion focus vs. prevention focus) were used to design the message content related to health. Natural sportscapes included grass, oceans, mountains, and other scenic environments. Urban sportscapes included floors, carpets, mirrors, concrete walls, and various indoor environments. In this study, the content of the promotion-focused messages and prevention-focused messages were modifications of the content developed by Higgins (1997). Promotion-focused messages depicted the pursuit of health through exercise, for example, “Practicing yoga helps improve the memory of older consumers.” Prevention-focused messages depicted that engaging in yoga is safe, for example, “Yoga is safe for elderlies.”

Three experts in the field of sports research were invited to be the evaluators. They had extensive knowledge of exercise management as well as experience in sports psychology and photographic techniques. The three expert evaluators started with 40 pictures, eliminating those with low-light, insufficient sharpness, and inappropriate perspectives. Finally, there were 16 photos selected for the experiment. The pictures used in the experiment are shown in Appendix 1.

#### Experimental Procedure

First, the participants were asked to confirm their health status before the experiment so as to ensure that they had no mental illnesses and met the measurement criteria for best corrected vision and visual perspective. Second, the researchers verbally communicated the experimental procedures to the participants. Third, in order to confirm that all the participants were volunteering to participate in the experiment and had normal cognitive abilities, each participant was asked to sign a consent form and take the Mini-Mental State Examination (MMSE). Participants with 9 years of education or more were required to score 24 points or higher, while participants with less than 9 years of education were required to score 16 points or higher in order to participate in the experiment. Fourth, the researcher verbally confirmed that the participant was physically able to engage in yoga. Because some older consumers who were willing to participate in the study indicated that they would be unable to do the postures shown in the pictures during the experimental process, they were disqualified. In this study, a total of 14 participants were excluded because they failed to pass the screening for the third and fourth steps mentioned above. Fifth, suitable participants were randomly assigned to each experimental condition.

Finally, a Tobii Pro X2-60 Eye Tracker (Tobii Technology, Inc., Stockholm, Sweden) was used to collect the eye movement data from the participants. The data were collected using specialized eye movement analysis software. The eye tracker was positioned in front of a 22-inch widescreen TFT monitor (1,920 × 1,080 pixels) to present the stimuli, and the test participant was asked to sit approximately 60 cm from the front of the screen monitor. Each experimental picture was displayed at random for 12 s. Immediately after displaying each image, the participant performed a behavioral intention assessment of the previous picture. The act of viewing an image and answering a behavioral intention question was marked as a set in each test. Between each set, the screen displayed a blank page for 1 s. The experimental study included a total of 16 sets, taking a total of 40–60 min.

### Results

#### The Characteristics of Participants

Therefore, 186 healthy individuals aged 50 or above who engaged in regular physical activities were recruited as participants. The characteristics of the participants are stated in Table 2.

**TABLE 2:** Characteristics of the older consumers.

	Mean	*SD*
**Gender**		
Male	68	
Female	118	
**Age (years)**	66.54	10.81
Male	63.13	9.94
Female	68.40	10.89
**MMSE**	26.80	3.86
Male	27.83	3.09
Female	26.24	4.14

*MMSE, Mini-Mental State Examination.*

#### Visual Attention of Older Consumers Toward Sports Marketing Pictures

Table 3 shows the results of a *t*-test used to analyze the differences in the fixation duration of the participants for each factor. First, with respect to the analytical results for the sportscapes, the average fixation count of the older participants on the natural sportscape pictures (mean nature sportscape = 246.65) was higher than that for the urban sportscape pictures (mean urban sportscape = 191.26). Furthermore, this difference was statistically significant [*t*(184) = 2.12, *p* = 0.04]. Thus, Hypothesis 1 was supported. Second, regarding the analytical results for the regulatory focus messages, the average fixation duration for the older participants on the promotion-focused messages (mean promotion focus = 221.79 ms) was higher than that on the prevention-focused messages (mean prevention focus = 154.93 ms). This difference reached a statistically significant level [*t*(184) = 2.14, *p* = 0.03]. However, in terms of visual attention toward the sportscape, the fixation duration of the participants on the prevention-focused messages (247.88 ms) was greater than that on the promotion-focused messages (191.35 ms). This difference also reached a statistically significant level [*t*(184) = 2.17, *p* = 0.02]. Thus, Hypothesis 3 was supported.

**TABLE 3:** The average fixation count (frequency) of eye movement under the stimulation conditions: message text and exercise location.

		Fixation count (frequency)								
		Message text			Exercise location			Total		
		Means	*t*	*P*	Means	*t*	*p*	Means	*t*	*p*
Stimulation condition	Natural sportscape (*n* = 94)	196.76	0.56	*p* = 0.53	246.65	2.12 *	*p* = 0.04	605.53	2.12	*p* = 0.07
	Urban sportscape (*n* = 92)	180.54			191.26			552.76		
	Promotion focus (*n* = 94)	221.79	2.14 *	*p* = 0.03	191.35	2.17 *	*p* = 0.02	569.81	2.17	*p* = 0.06
	Prevention focus (*n* = 92)	154.93			247.88			589.33		

*p < 0.05.

Furthermore, through the ANOVA analysis, the differences in the fixation duration of older consumers for conditions A, B, C, and D were tested (see Table 4). The results showed statistically significant differences in the fixation duration of each group [*F*(3, 182) = 3.06, *p* = 0.02). The results of the comparative analysis showed that an urban sportscape paired with a promotion-focused message (Condition C) was less likely to attract the visual attention of older consumers than other three conditions. To distinguish the attractiveness of these three conditions to older consumers, following, the heat maps were used to analyze the effects of different conditions on visual attention and an ANOVA was used to analyze the effects of different conditions on behavioral intentions.

**TABLE 4:** The average fixation duration (ms) of eye movement performance under the four conditions.

Means					
A condition (*n* = 46)	B condition (*n* = 48)	C condition (*n* = 48)	D condition (*n* = 44)	*F*	*Post hoc*
570.45 ms	601.76 ms	472.45 ms	582.50 ms	3.06*	A, B, D > C

*p < 0.05.

The different visual responses of the older consumers to the sports marketing pictures are shown in Table 5. Compared to the sports marketing pictures of an urban sportscape (C and D), the pictures of a natural sportscape (A and B) had larger, more colorful shaded areas. However, the prevention-focused images (B and D) had slightly darker and redder shaded areas than the promotion focus images (A and C).

**TABLE 5:** Heat maps.

Factors		Regulatory-focused messages	
		Promotion focus	Prevention focus
Sportscapes	Natural sportscape	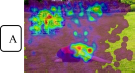	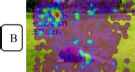
	Urban sportscape	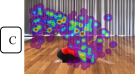	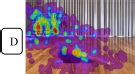

#### Exercise-Related Behavioral Intentions of Older Consumers

A *t*-test analysis was used to explore the effects that sportscapes and regulatory-focused messages had on the behavioral intentions of the participants. First, the effects of the different sportscapes on behavioral intentions reached a significant level [*t*(184) = 3.46, *p* < 0.001]. Thus, Hypothesis 2 was supported. Second, the *t*-test results indicated that the effects of promotion-focused messages and prevention-focused messages on behavioral intention were not statistically significant (*p* = 0.07). Thus, Hypothesis 4 was not supported.

Furthermore, an ANOVA was used to analyze the effects of the different conditions on the behavioral intentions of the participants. As shown in Table 6, the effects of all the conditions on the five behavioral intentions were as follows: BI1 [*F*(3, 182) = 15.98, *p* < 0.001], BI2 [*F*(3, 182) = 6.98, *p* < 0.001], BI3 [*F*(3, 182) = 5.82, *p* < 0.001], BI4 [*F*(3, 182) = 6.81, *p* < 0.001], and BI5 [*F*(3, 182) = 4.04, *p* = 0.007] were statistically significant. The results showed that images that combine natural sportscapes and prevention-focused messages generate the highest level of behavioral intention from consumers.

**TABLE 6:** Behavioral intention of older consumers under the four conditions.

BI items	Means				*F*	*Post hoc*
	A condition	B condition	C condition	D condition		
BI 1	4.33	4.41	3.50	3.20	15.98*	A, B > C, D
BI 2	3.95	4.32	3.32	3.35	6.98*	B > C, D
BI 3	4.24	4.27	3.64	3.60	5.82*	A, B > C, D
BI 4	4.00	4.41	3.54	3.65	6.81*	B > C, D
BI 5	4.19	4.32	3.73	3.65	4.04*	B > D

BI, behavioral intention. *p < 0.05.

## Study 2

Study 2 examined whether compatibility between regulatory focus and sportscapes influences consumers’ online purchase choices of sports programs. A 2 regulatory focus (promotion focus vs. prevention focus) × 2 sportscape (natural vs. urban) between-subjects experimental design was employed.

### Methods

#### Experimental Procedure

Participants were recruited from the Taichung Senior Citizens Association in Taiwan for Study 2, with a final usable sample of 75 and a response rate of 95.43% (Age *M* = 66.7 years; Female: 63.5%). Since Study 1 demonstrated that sportscapes and regulatory focus messages are both key factors that affect visual attention and behavioral intention, Study 2 was specifically focused on the impact of sportscapes and regulatory focus messages on purchase choices. The research methods and processes of Study 2 were similar to those used in Study 1.

**FIGURE 2:**
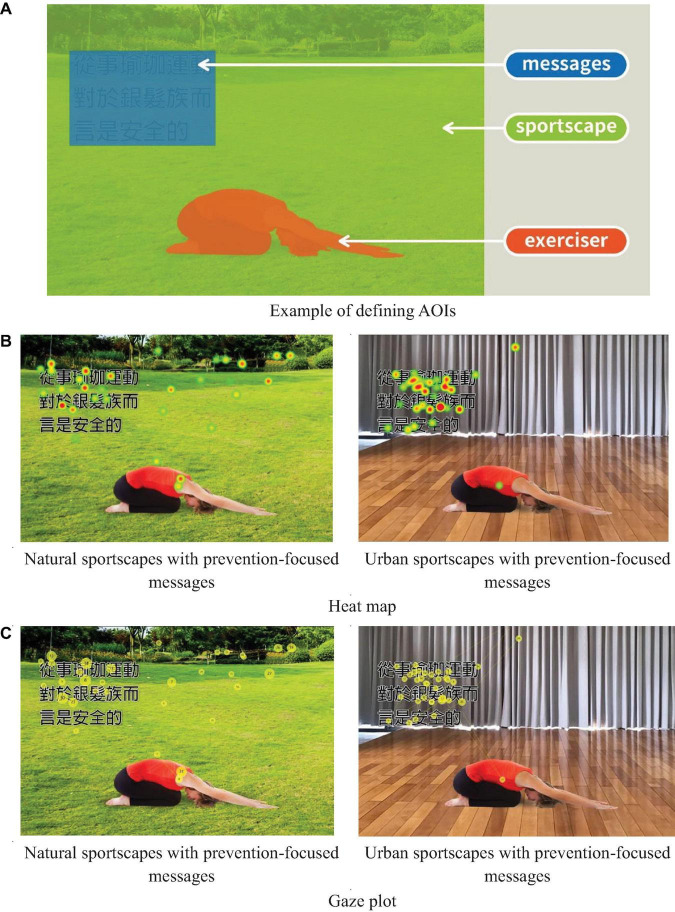
Examples of an eye tracking data analysis for a sports marketing image. Adapted from Freepik Images.

Since eye movement distance is short, rapid, and difficult to capture, to measure visual attention, the wellness service image was divided into three areas of interest (AOI) (Figure 2), including Exerciser, Messages, and Sportscape. This method was used to confirm which part of the sports marketing image was the most interesting to the observers (Figure 2A). Eye tracking technology was used to visualize the participants’ visual attention in the form of a heat map and gaze plot (Figures 2B,C), allowing researchers to explore the degree of visual attention.

As the participants scanned randomly assigned exercise marketing images, an eye tracker recorded individual eye tracking data (e.g., gaze and fixations). The participants were told that they had a sufficient budget to purchase the exercise courses. This eliminated budgetary pressure related to the purchase. The price of each exercise plan was not included in the advertisement since this could interfere with the exercise service selection process. After observing one of the four groups of marketing advertisements, the participants answered whether they would choose or not choose to participate in such sports programs.

### Results

A one-way ANOVA was used to evaluate the differences among the buying decisions under conditions involving four different forms of sports advertising. According to the different combinations of online advertising images, there were obvious differences in the amount of purchase decisions triggered. Overall, 63, 59, 43, and 39% of the participants were more willing to choose exercise programs with natural sportscapes emphasizing prevention-focused messages, urban sportscapes emphasizing prevention-focused messages, a natural sportscape emphasizing promotion-focused messages, and an urban sportscape emphasizing promotion-focused messages, respectively.

The ANOVA analysis results showed that the fixation duration of consumers observing the four sports marketing images was significantly different among the four conditions taken into account during the study [*F*(3, 74) = 8.341; *p* < 0.001] (Table 7). The Tukey *post-hoc* test revealed that the fixation duration on the B image [8843.67, 95% CI (5205.71, 12481.62); *p* < 0.001] was higher than the fixation duration on the D image [3308.67, 95% CI (2420.65, 4196.68); *p* < 0.001], the A image [3217.67, 95% CI (2640.68, 3794.65); *p* < 0.001], and the C image [2790.33, 95% CI (1811.79, 3768.87); *p* < 0.001]. Thus, hypotheses 3 and 6 were supported.

**TABLE 7:** The average fixation duration (ms) of eye movement under the four conditions for the sports marketing images.

Means					
Natural sportscapes		Urban sportscape			
Promotion- focused	Preventive- focused	Promotion- focused	Preventive- focused	F	*Post hoc*
	
A (*n* = 18)	B (*n* = 21)	C (*n* = 18)	D (*n* = 18)		
3217.66 ms	8843.67 ms	2790.33 ms	3308.66 ms	8.341*	B > D, A, C

*p < 0.05.

In addition, the results of the ANOVA analysis shown in Table 8 indicated that the fixation durations of consumers observing the sports marketing images were significantly different among the three AOIs (Exerciser, Messages, and Sportscape) taken into account during the study [*F*(2, 74) = 4.283; *p* < 0.01]. The Tukey *post-hoc* test revealed that the fixation duration on the Sportscape image [5441.56, 95% CI (5111.72, 5771.40); *p* < 0.001] was higher than that on Messages image [6267.36, 95% CI (2836.10, 9698.62); *p* < 0.001], as well as that on the Exerciser image [2427.76, 95% CI (2640.68, 3794.65); *p* < 0.001].

**TABLE 8:** Total average duration of fixation on the different AOIs among the four conditions in the sports marketing images.

AOIs			*F*	*Post hoc*
A: Exerciser	B: Messages	C: Sportscape		
2427.76 ms	5441.56 ms	6267.36 ms	4.283**	C > A, B B > A C > A

**p < 0.001.

**FIGURE 3:**
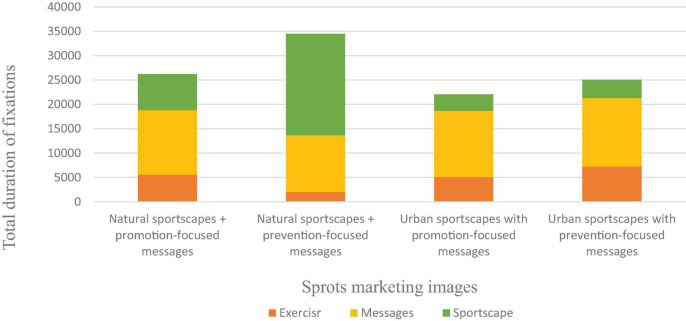
Total average duration of fixation on the different AOIs among the four types of sports marketing images.

The results for the heat map shown in Figure 2B and those for the gaze plot shown in Figure 2C indicated that in natural sportscapes, the consumers first observed the natural sportscapes, and then read the messages. In the urban sportscapes, consumers read the messages first, and then observed the sportscapes. In addition, the results for the consumers’ choices showed that the advertisements most of interest to the consumers occurred under the B conditions, followed by A, D and C. As shown in Figure 3, the results of heat map showed that a natural sportscape was the area of greatest interest for the observers. The natural sportscape had a red hotspot ratio that was higher than the other AOIs. Figure 3 illustrates these results, where the results of the gaze plot show that all sports marketing images exhibited the same phenomenon, especially, sportscape areas of natural sportscape with preventive focus messages get relatively higher percentage of total average duration of fixation. A natural sportscape attracts the viewer for a longer fixation duration. The visual appeal of a natural sportscape is maximized in natural sportscape with preventive focus messages.

## Discussion

The research results showed that promoting the benefits of exercising in a natural sportscape with prevention-focused messages may be an effective way to encourage online exercise consumption. To the best of our knowledge, this research is one of the first attempts to evaluate consumer decision-making processes in the context of online exercise marketing by using eye-tracking technology. This study contributes to researchers and managers addressing sports marketing in the following ways:

First, the results shown that images of exercise in natural sportscapes can arouse the behavioral intention of older consumers toward engaging in exercise. The images of exercise in natural sportscapes successfully promoted the behavioral intention of older consumers to engage in yoga. It is worth noting that the results of the questionnaire survey in this study confirmed that images with natural environment cues not only sparked the interest of the older consumers in yoga-related exercise, but also led to positive emotions. In addition, people engaging in exercise may be easily stimulated by external advertising, which can lead them to consider exercise. This study showed that older consumers, after observing images with natural environments in the background, were more interested in participating in yoga-related physical activities and observing more images related to yoga than was the case after they observed images with urban sportscapes as the background. This result is consistent with the findings of Calogiuri and Elliott (2017) suggesting that older consumers prefer natural environments as their exercise venues. These results imply that natural sportscapes can provide more mental health benefits to older consumers than urban sportscapes. By designing natural exercise images (e.g., exercising on grass or wooden floors) that lead to more mental health benefits to older consumers, sports marketers are stimulating them to consume natural exercise options.

Second, the result of the ANOVA on the eye-tracking data showed that the utilization of any natural environment as a site for sports services contributes to attracting the visual attention of older consumers since natural sportscapes attract the visual attention of older consumers. Older consumers show a high degree of preference for natural sportscapes as sites for exercise. The findings of this study confirm that natural sportscapes are an important factor in terms of attracting the visual attention of older consumers. Specifically, the fixation duration of the older consumers on natural sportscapes was at least 75% higher than that on urban sportscapes. This may have been the result of the mental health benefits of the natural environment, where the clues conveyed by the natural sportscape allow the observers to relax, which in turn reduced their perceived levels of stress (Ulrich et al., 1991; Losada et al., 2017; Wang et al., 2019b,^2020^). Therefore, consumers are willing to spend more time observing pictures showing the natural environment. These results echo the findings of Losada et al. (2017), who suggest that natural environments are more likely to be preferred by older consumers. This result is also similar to those obtained in a previous study by Prothero and Fitchett (2000), who suggested that service providers/marketing managers can serve as advocates for environmental issues and can redesign marketing images to bring about more consumption. To provide sports services that meet the expectations of older consumers, sports firms should choose pictures of natural environments (such as green forests or green grasslands) as the background when designing images encouraging exercise consumption.

Third, the eye-tracking data also confirmed that the fixation duration of older consumers on natural sportscapes was higher than that on urban sportscapes, and their fixation duration on prevention-focused messages was higher than that on promotion-focused messages. In addition, based on the heat maps, it was found that images combining natural sportscapes with prevention-focused messages were most likely to receive higher visual attention. In contrast, images that combined urban sportscapes with promotion-focused messages received the least visual attention. This means that the interaction between the characteristics of a sportscape and the meaning of a sports marketing message can affect the sports decisions of older consumers. When a picture presents sportscapes and sports marketing messages, older consumers will consider whether the sportscape is suitable for their specific physical conditions and therefore attractive to them and whether the sports marketing message reflects their needs. Because older consumers are physically less agile and quick, they are concerned about the safety of any sportscape, which is their primary consideration when engaging in exercise (Losada et al., 2017). These results echo the findings of Wu et al. (2016). Their studies showed that safety was the most important factor for older consumers in terms of assessing whether to engage in physical exercise. Therefore, effective use of visual features (e.g., natural sportscapes with prevention-focused strategies) will encourage customers to spend the least energy on processing information related to the benefits of exercise (Avnet and Higgins, 2006; Micu and Chowdhury, 2010) as compared to other exercise program options, and they will in turn choose exercise program options that have most potential to promote their mental and physical health.

## Conclusion

It was found in the current study that sports marketing images of natural sportscapes with prevention-focused messages attracted more visual attention and greater behavioral intention leading to consumption choices in older customers than the other sports marketing images. The findings of this study reveal the importance of paying attention to the needs of older consumers. To affect the exercise consumption of older consumers, sports firms must first draw their visual attention.

### Practical Implications

Suggestions for wellness service firms or sports firms in terms of designing sports marketing pictures are as follows: First, to encourage older consumers to engage in exercise, when designing sports marketing pictures, sports firms should consider provide a message about a natural sportscape (i.e., turf, parks, or forest) and should suggest safety benefits that will meet the needs of older consumers engaging in exercise. Second, by designing marketing images with natural sportscapes, sports service providers may attract consumers’ visual attention and influence their subsequent behavioral intentions and choices.

### Limitations and Suggestions for Future Research

This study had the following limitations: First, during the experiment, some older consumers over the age of 80 indicated that they were not able to perform the assigned yoga positions. Thus, the participants could not be tested. In the future, when conducting an experiment focused on yoga, it is necessary to consider whether the older consumers are able to do the yoga positions. Second, this study used cross-sectional data to explore the hypothesized relationships. To avoid common method variations, future studies could employ a longitudinal design to collect data for the purpose of exploring the process by which visual attention produces actual online consumption behavior. Finally, observing the text in a message drains consumers’ cognitive resource. In order to avoid the excessive complexity of the text in the messages, which consumes too much cognitive resources, and reduces the effectiveness of the messages. By divided the source code into different AOIs, the source code analysis with eye tracking technology can provide more accurate and precise results (Katona, 2021). Future research can measure cognitive load using eye-tracking parameters (Katona, 2022) to ensure that the content of messages does not cause excessive cognitive load on consumers.

## Data Availability Statement

The datasets presented in this article are not readily available because the authors do not have the authority to share the dataset. Requests to access the datasets should be directed to Banson, a9125447@hotmail.com.

## Ethics Statement

The studies involving human participants were reviewed and approved by the National Cheng Kung University’s Ethics Committee. The patients/participants provided their written informed consent to participate in this study.

## Author Contributions

T-CW: conceptualization, formal analysis, investigation, and writing—original draft. T-WT: investigation, formal analysis, and writing—original draft. C-LT: methodology, validation, writing—review and editing. All authors contributed to the article and approved the submitted version.

## Conflict of Interest

The authors declare that the research was conducted in the absence of any commercial or financial relationships that could be construed as a potential conflict of interest.

## Publisher’s Note

All claims expressed in this article are solely those of the authors and do not necessarily represent those of their affiliated organizations, or those of the publisher, the editors and the reviewers. Any product that may be evaluated in this article, or claim that may be made by its manufacturer, is not guaranteed or endorsed by the publisher.

## Appendix

**APPENDIX 1:**

Factors		Regulatory-focused messages	
		Promotion focus	Prevention focus
Sportscapes	Nature sportscape		
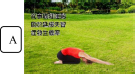
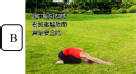
	Urban sportscape		
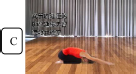
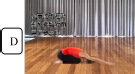

*Text in the Promotion Focus message: Practicing yoga can help reduce the risk of dementia. Text in the Prevention Focus message: Yoga is safe for elderlies.*
